# The Impact of ExoS on *Pseudomonas aeruginosa* Internalization by Epithelial Cells Is Independent of *fleQ* and Correlates with Bistability of Type Three Secretion System Gene Expression

**DOI:** 10.1128/mBio.00668-18

**Published:** 2018-05-01

**Authors:** Abby R. Kroken, Camille K. Chen, David J. Evans, Timothy L. Yahr, Suzanne M. J. Fleiszig

**Affiliations:** a School of Optometry, University of California, Berkeley, Berkeley, California, USA; b College of Pharmacy, Touro University California, Vallejo, California, USA; c Department of Microbiology and Immunology, University of Iowa, Iowa City, Iowa, USA; d Graduate Groups in Vision Science, Microbiology, and Infectious Diseases & Immunity, University of California, Berkeley, Berkeley, California, USA; Georgia Institute of Technology School of Biological Sciences

**Keywords:** bistability, ExoS, FleQ, *Pseudomonas aeruginosa*, epithelial cells, host cell invasion, intracellular bacteria, type three secretion system

## Abstract

Pseudomonas aeruginosa is internalized into multiple types of epithelial cell *in vitro* and *in vivo* and yet is often regarded as an exclusively extracellular pathogen. Paradoxically, ExoS, a type three secretion system (T3SS) effector, has antiphagocytic activities but is required for intracellular survival of P. aeruginosa and its occupation of bleb niches in epithelial cells. Here, we addressed mechanisms for this dichotomy using invasive (ExoS-expressing) P. aeruginosa and corresponding effector-null isogenic T3SS mutants, effector-null mutants of cytotoxic P. aeruginosa with and without ExoS transformation, antibiotic exclusion assays, and imaging using a T3SS-GFP reporter. Except for effector-null PA103, all strains were internalized while encoding ExoS. Intracellular bacteria showed T3SS activation that continued in replicating daughter cells. Correcting the *fleQ* mutation in effector-null PA103 promoted internalization by >10-fold with or without ExoS. Conversely, mutating *fleQ* in PAO1 reduced internalization by >10-fold, also with or without ExoS. Effector-null PA103 remained less well internalized than PAO1 matched for *fleQ* status, but only with ExoS expression, suggesting additional differences between these strains. Quantifying T3SS activation using GFP fluorescence and quantitative reverse transcription-PCR (qRT-PCR) showed that T3SS expression was hyperinducible for strain PA103Δ*exoUT* versus other isolates and was unrelated to *fleQ* status. These findings support the principle that P. aeruginosa is not exclusively an extracellular pathogen, with internalization influenced by the relative proportions of T3SS-positive and T3SS-negative bacteria in the population during host cell interaction. These data also challenge current thinking about T3SS effector delivery into host cells and suggest that T3SS bistability is an important consideration in studying P. aeruginosa pathogenesis.

## IMPORTANCE

P. aeruginosa is often referred to as an extracellular pathogen, despite its demonstrated capacity to invade and survive within host cells. Fueling the confusion, P. aeruginosa encodes T3SS effectors with anti-internalization activity that, paradoxically, play critical roles in intracellular survival. Here, we sought to address why ExoS does not prevent internalization of the P. aeruginosa strains that natively encode it. Results showed that ExoS exerted unusually strong anti-internalization activity under conditions of expression in the effector-null background of strain PA103, often used to study T3SS effector activity. Inhibition of internalization was associated with T3SS hyperinducibility and ExoS delivery. PA103 *fleQ* mutation, preventing flagellar assembly, further reduced internalization but did so independently of ExoS. The results revealed intracellular T3SS expression by all strains and suggested that T3SS bistability influences P. aeruginosa internalization. These findings reconcile controversies in the literature surrounding P. aeruginosa internalization and support the principle that P. aeruginosa is not exclusively an extracellular pathogen.

## INTRODUCTION

Pseudomonas aeruginosa is an opportunistic Gram-negative bacterial pathogen capable of colonizing mucosal epithelia of immunocompromised hosts and spreading systemically. Commonly infected sites include burn wounds, lung epithelia in acute pneumonia or cystic fibrosis patients, and epithelial surfaces in contact with catheters, ventilators, or contact lenses (1, 2). Studies of P. aeruginosa pathogenesis in model systems of different target tissues have demonstrated that the type three secretion system (T3SS) plays major roles in acute infection outcomes (3). The T3SS is a molecular syringe capable of delivering protein effectors across host cell membranes. The following four T3SS-secreted effectors have been discovered for P. aeruginosa: ExoU (a phospholipase), ExoS and ExoT (bifunctional enzymes with Rho-GTPase activating protein [RhoGAP] and ADP-ribosyltransferase [ADP-r] domains), and ExoY (a nucleotidyl cyclase) (4–8). Most strains encode ExoT and ExoY, but ExoU and ExoS are mostly mutually exclusive in individual strains (9, 10). Very few strains encode all four effectors. These effectors are activated by a mammalian host cell cofactor(s) thought to limit their catalytic activity to the host cytosol (5, 11–13). Rather than exhibiting constitutive expression, the T3SS is induced either under low-calcium conditions or by host cell contact (14, 15).

While often regarded as an extracellular pathogen, P. aeruginosa can invade multiple types of epithelial cell *in vitro* and *in vivo*. *In vivo*, this was first illustrated by our group using transmission electron microscopy to show bacteria within the cytoplasm of epithelial cells during corneal infections in mice (16). This was later demonstrated *in vitro* for corneal epithelial cells using gentamicin protection assays (17). Multiple research groups have since studied P. aeruginosa internalization mechanisms, showing involvement of the growth phase using HeLa cells (18), the cystic fibrosis transmembrane-conductance receptor in airway epithelial cells (19), lipid zippering in MDCK cells (20), lipid raft internalization (also in corneal epithelial cells) (21), and type 6 secretion system-delivered effectors that mobilize microtubules to trigger uptake (also using HeLa cells) (22).

However, our published data have also shown variability in internalization capacity among clinical isolates (23). Those less able to invade tended to have acute cytotoxic activity, leading us to propose classification of P. aeruginosa isolates as either cytotoxic or invasive strains based on their interactions with epithelial cells. Further investigation showed that these alternate phenotypes relate directly to the different T3SS effectors encoded, with cytotoxic strains encoding ExoU and not ExoS and the reverse for invasive strains (10).

Both ExoS and ExoT destabilize the actin cytoskeleton through RhoGAP activity (24–27) by prematurely catalyzing hydrolysis of GTP to GDP in Rho, Rac, and Cdc42, thereby inhibiting actin-based internalization (10, 28). Given that ExoS and ExoT are capable of anti-internalization activities, it appears paradoxical that both effectors are encoded by P. aeruginosa strains that invade cells. Additionally, our published data show that ExoS ADP-r activity is, in fact, required for avoidance of lysosomal degradation in corneal and bronchial epithelial cells after invasion (29) and via separate mechanisms to generate membrane blebs inside these cells that are subsequently used as an intracellular replicative niche (30, 31). ExoS-induced bleb niches are mechanistically distinct from apoptotic blebs in both corneal and bronchial epithelial cells and are acted upon by osmotic forces (32).

Much of our current understanding of ExoS antiphagocytic activity was derived via examination of ectopic expression without bacteria or in *trans* expression in the background of an effector-null mutant of strain PA103, a cytotoxic strain that does not natively encode ExoS. Here, we used live-cell imaging and a T3SS reporter to explore how invasive strains such as PAO1 are internalized while still being able to express and secrete this antiphagocytic effector.

## RESULTS

### P. aeruginosa occupies bleb niches in HeLa cells.

**FIG 1:**
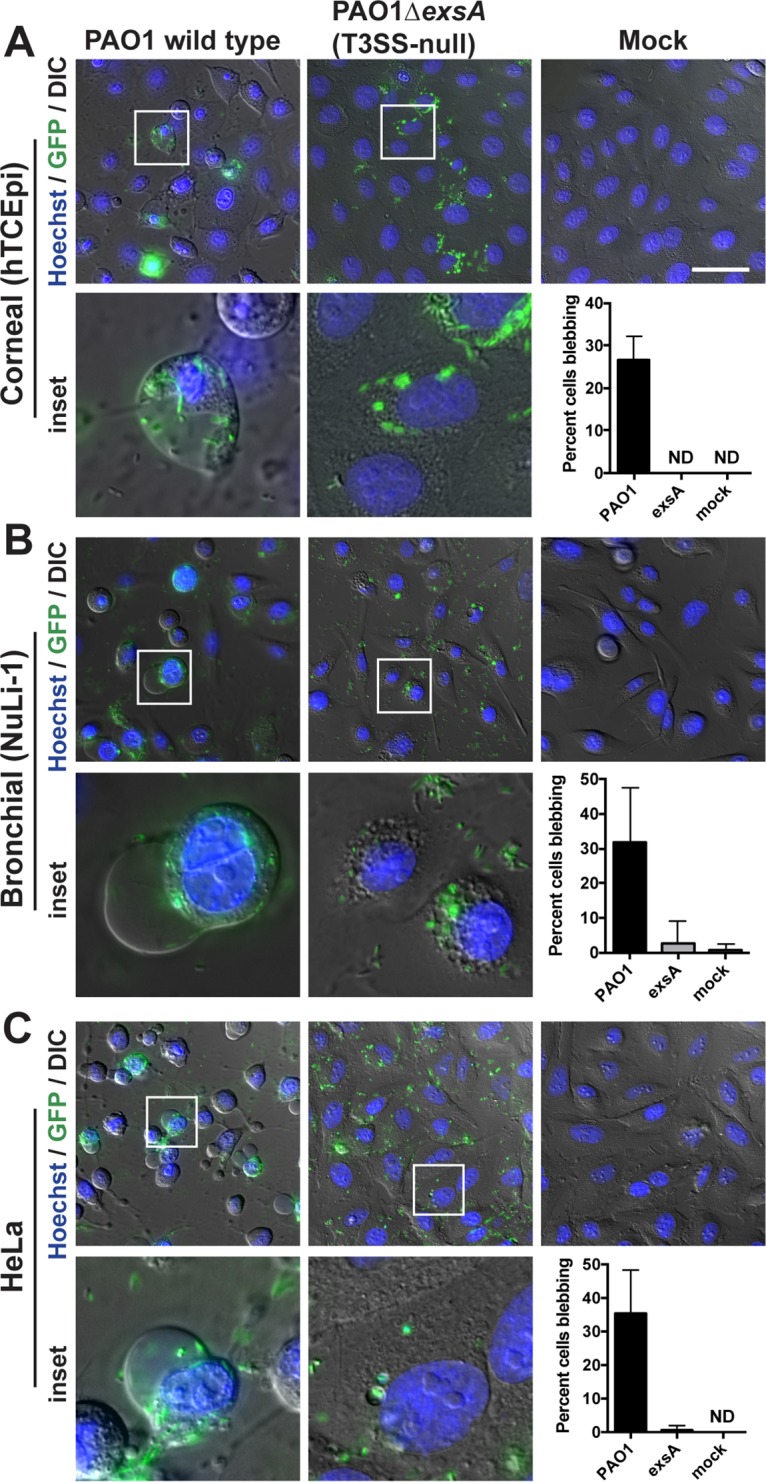
P. aeruginosa intracellular survival and generation of bleb niches in HeLa cells. (A) Human corneal epithelial cells (hTCEpi), (B and C) Bronchial epithelial cells (NuLi) (B) or HeLa cells (C) were infected with PAO1 or an *exsA* mutant expressing GFP constitutively (plasmid pSMC2). Amikacin (200 µg/ml) was added 3 h postinfection to eliminate extracellular bacteria. Cells were imaged at 7 h postinfection. Results of quantification of blebbing cells from approximately 200 cells per experiment are shown. ND, none detected. Bar, 50 µm.

ExoS prevents P. aeruginosa internalization into HeLa cells (33, 34) but is required for intracellular survival in corneal and bronchial epithelial cells (31, 32). To reconcile these findings, we hypothesized that differences in epithelial cell types explained these apparently contradictory outcomes. Differentiated human telomerase-immortalized corneal epithelial cells (hTCEpi), bronchial epithelial cells (NuLi-1), and HeLa cells were compared for infection with green fluorescent protein (GFP)-expressing P. aeruginosa strain PAO1 and a T3SS (*exsA*) mutant, both expressing constitutive GFP from plasmid pSMC2. While HeLa cells infected with PAO1 were rounded as previously reported (34, 35), the presence or absence of rounding differed for corneal and bronchial epithelial cells, suggesting reduced susceptibility to T3SS effector delivery or cytoskeleton-disruptive effects (Fig. 1). Nevertheless, intracellular bacteria were present in all 3 cell types, many within bleb niches, as previously shown for corneal and bronchial epithelial cells (30, 32). Consistent with published data for other cell types, HeLa cell bleb niches were T3SS dependent.

### P. aeruginosa within HeLa cells are T3SS positive.

To understand why P. aeruginosa strain PAO1 invades HeLa (and other) cells while natively expressing effectors capable of anti-internalization activity, we next investigated the timing and localization of T3SS activation during HeLa cell infection using T3SS reporter pJNE05 (29, 35). With this reporter, the level of fluorescence of T3SS-positive bacteria was previously found to be significantly higher than that seen with T3SS-negative bacteria by fluorescence-activated cell sorting (35). That result was confirmed here using quantitative image analyses. The level of fluorescence measured for T3SS-positive bacteria was ~6-fold higher than that measured for T3SS-negative bacteria (see Fig. S1 in the supplemental material).

10.1128/mBio.00668-18.1 FIG S1  Average fluorescence intensities of T3SS-positive and -negative bacteria. T3SS-negative or -positive bacteria (20 in each group) from two independent confocal microscopy images of invaded cells without antibiotics were measured for average GFP intensity, and the results were compared using an unpaired *t* test. Download FIG S1, TIF file, 2.1 MB. Copyright © 2018 Kroken et al. 2018 Kroken et al. This content is distributed under the terms of the Creative Commons Attribution 4.0 International license.

**FIG 2:**
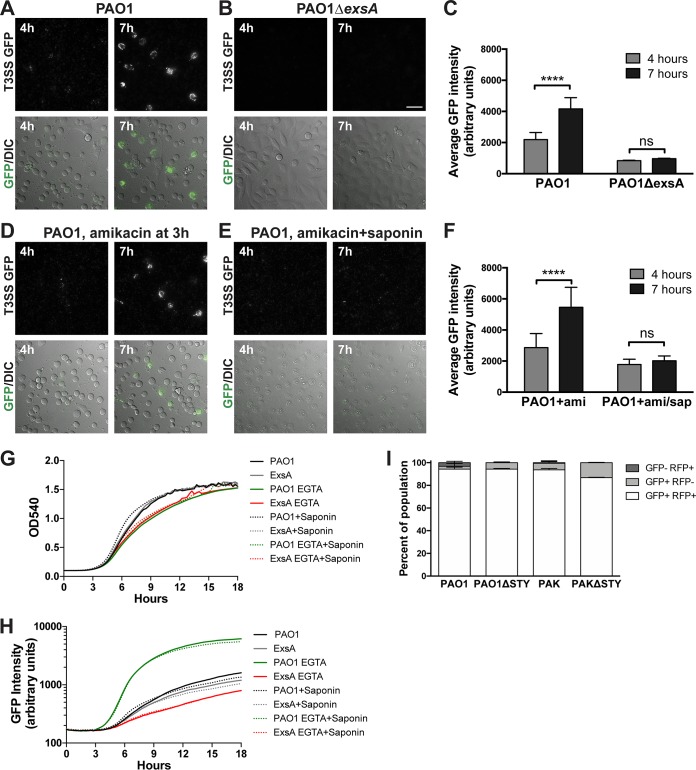
Intracellular P. aeruginosa cells are T3SS positive. (A and B) HeLa cells were infected with strain PAO1 (A) or mutant PAO1Δ*exsA* (T3SS-null) (B), both transformed with T3SS-GFP reporter pJNE05. Cell-associated T3SS-positive bacteria were observed accumulating at between 4 and 7 h postinfection. Bar, 50 µm. (C) The average GFP intensity of cell-associated bacteria was quantified, and the data in the indicated columns were compared using an unpaired *t* test. (D) Infected cells were treated with amikacin (200 µg/ml) at 3 h postinfection, and accumulation of T3SS-positive bacteria was observed. (E) Infected cells were treated with amikacin and 0.1% saponin to permeabilize cell membranes as a control. (F) Data were quantified as described for panel C. (G and H) Bacterial growth in TSB–100 mM glycine–1% glycerol containing 2 mM EGTA or 0.1% saponin, showing no effect on P. aeruginosa viability (G) or the capability of T3SS induction measured by the reporter (H). (I) HeLa cells were also infected with the indicated P. aeruginosa strains and T3SS mutants transformed with both the T3SS-GFP reporter and p67T1 (dTomato) as described above, and amikacin (200 µg/ml) was added at 3 h postinfection. At between 6 and 12 h postinfection, the total population of HeLa cells containing internalized bacteria was determined via constitutive dTomato expression, and the percentages of HeLa cells containing GFP (T3SS)-positive bacteria were also determined. The majority of HeLa cells containing dTomato-positive intracellular bacteria also contained GFP (T3SS)-positive bacteria.

PAO1 infection of HeLa cells was associated with low GFP (T3SS) expression at 4 h postinfection; the level was found to have increased significantly in cell-associated bacteria by 7 h postinfection (Fig. 2A and C). Little or no GFP signal was observed with infection performed using an *exsA* mutant, consistent with the presence of the *exsA*-responsive promoter in the T3SS reporter (Fig. 2B and C). A live time-lapse movie was produced with images taken every 15 min for 5 h using the conditions described for panel A. The results showed that T3SS-positive bacteria remained confined to the boundaries of HeLa cell borders over time (see Movie S1 in the supplemental material).

10.1128/mBio.00668-18.3 MOVIE S1  Time-lapse video microscopy of P. aeruginosa strain PAO1 infection of HeLa cells. Cells were infected with PAO1 expressing T3SS-GFP reporter pJNE05. Imaging began immediately and continued for 5 h at 10-min intervals. No antibiotics were added in this time-lapse imaging experiment. Download MOVIE S1, MOV file, 19.9 MB. Copyright © 2018 Kroken et al. 2018 Kroken et al. This content is distributed under the terms of the Creative Commons Attribution 4.0 International license.

To determine the contribution of intracellular bacteria to the total T3SS-induced population, extracellular bacteria were killed 3 h postinfection using amikacin, a non-cell-permeative antibiotic. Amikacin efficacy against bacteria harboring pJNE05 (encodes gentamicin resistance) was confirmed in control experiments (not shown). GFP (T3SS) expression continued after amikacin treatment, with many more GFP-positive, cell-associated bacteria present at 7 h than at 4 h. Since non-cell-permeative amikacin was present between these time points, the increased number of bacteria/GFP signal at 7 h suggested that T3SS gene expression occurred during intracellular bacterial replication, as extracellularly induced bacteria could not have entered the HeLa cells during this time (Fig. 2D and F). Moreover, GFP expression was no longer observed at 7 h when saponin (0.1%) was added with amikacin at 3 h to permeabilize host cell membranes and to allow antibiotic access to intracellular bacteria; that result represents further evidence that these bacteria were intracellular (Fig. 2E and F). Results of *in vitro* control experiments performed without epithelial cells confirmed that saponin (0.1%) had little effect on P. aeruginosa growth (Fig. 2G) or EGTA-induced T3SS reporter expression (Fig. 2H). Other controls (not shown) confirmed that amikacin (200 µg/ml) killed bacteria even at 10-fold-higher levels than the inocula used and showed no visible evidence of the presence of biofilms that might resist antibiotic killing.

We next explored whether all observed intracellular populations of P. aeruginosa were T3SS positive by the use of strain PAO1 and the corresponding effector mutant, strain PAO1Δ*exoSTY*, transformed with both the T3SS reporter and p67T1 (expresses dTomato constitutively) (36). Time-lapse images were obtained between 6 h and 12 h postinfection, with amikacin added at 3 h to exclude extracellular bacteria (Fig. 2I). Most HeLa cells contained intracellular bacteria that were both dTomato positive and GFP positive, consistent with T3SS expression by most intracellular bacteria. Very few examples of dTomato-positive and GFP-negative bacterial populations were detected inside HeLa cells. Similar results were obtained with another invasive strain, PAK (Fig. 2I). Some intracellular bacteria were GFP positive and dTomato negative, suggesting plasmid loss.

**FIG 3:**
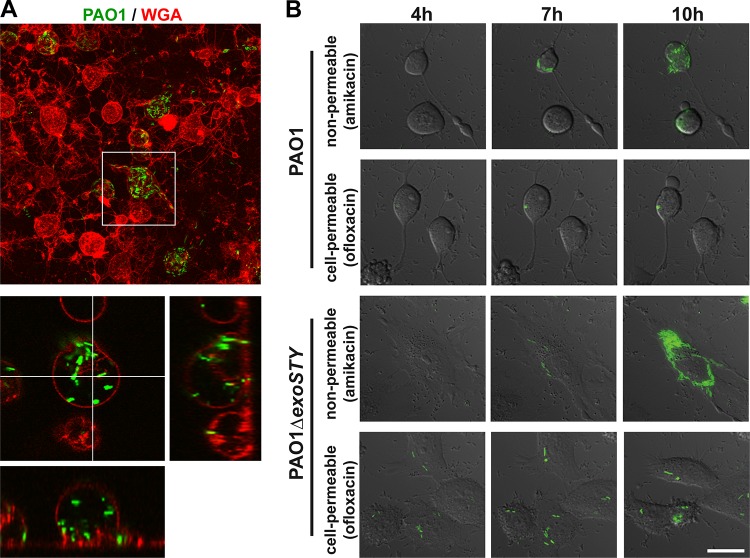
Ofloxacin treatment limits the intracellular location of P. aeruginosa PAO1 and its effector-null mutant. (A) HeLa cells were infected with strain PAO1 transformed with T3SS-GFP reporter pJNE05. Extracellular bacteria were killed with 200 µg/ml amikacin at 3 h, and WGA-Alexa Fluor 647 was added for 15 min and removed prior to imaging. The maximum projection is shown along with a single slice with XZ and YZ projections. (B) HeLa cells were infected with strain PAO1 or mutant PAO1Δ*exoSTY* transformed with pJNE05. Extracellular bacteria were killed with 200 µg/ml amikacin at 3 h, and all bacteria were killed with amikacin in combination with 25 µg/ml ofloxacin, which is cell permeative. Imaging began at 4 h postinfection and continued to be performed at 1-h intervals until 16 h. Selected intervals are shown. Bar, 25 µm.

Intracellular location of T3SS-positive PAO1 in HeLa cell bleb niches was further verified using two approaches. First, wheat germ agglutinin (WGA) was used to label the plasma membrane of infected HeLa cells. Confocal imaging showed bacteria enclosed within the plasma membrane (Fig. 3A). Second, amikacin (non-cell permeative) was compared to ofloxacin (cell permeative; also kills intracellular bacteria). Ofloxacin, but not amikacin, suppressed the appearance of additional GFP-expressing bacteria above the baseline numbers present when the antibiotic was added (Fig. 3B). Unexpectedly, the T3SS effector null PAO1Δ*exoSTY* mutant also replicated intracellularly in HeLa cells (Fig. 3B), which it does not do in corneal epithelial cells, for which ExoS is required (30). Notably, GFP-positive bacteria remained visible for several hours (i.e., they did not lyse and release GFP) following exposure to ofloxacin, consistent with its action as a DNA gyrase inhibitor. This differs from the activity of amikacin and gentamicin, which inhibit protein synthesis and result in bacterial lysis and loss of GFP detection shortly after exposure.

### The impact of ExoS on internalization varies among P. aeruginosa isolates.

Having established that P. aeruginosa strain PAO1 can use ExoS to form bleb niches in HeLa cells, as in other epithelial cell types, and that intracellular bacteria and their daughter cells can be T3SS positive, we explored whether the reported discrepancies with respect to the efficiency of anti-internalization resulting from the presence of ExoS are related to differences among strains.

Cytotoxic strain PA103 has been used widely for exogenous ExoS expression. However, PA103 is unusual in that it is aflagellate (*fleQ* mutant), and it natively encodes only two of four known T3SS effectors (ExoU and ExoT) (33, 37, 38). While effector-null mutants of PA103 are internalized, similarly to other P. aeruginosa strains (28, 30), expression of ExoS (or ExoT) in *trans* enables strong anti-internalization activity such that very few bacteria enter HeLa cells (28, 33). Thus, PA103 effector-null mutants have been an excellent tool for delivering ExoS (or ExoT) into HeLa cells to study impact without the added complexity presented by intracellular bacteria (39).

Since PA103 natively encodes ExoU, not ExoS, we tested the hypothesis that cytotoxic strains differ from invasive strains in how ExoS impacts their internalization. HeLa cells were infected with several laboratory strains of P. aeruginosa, including the following: PAO1 and PAK (invasive; natively encode ExoS, ExoT, and ExoY), PA14 (cytotoxic; natively encodes ExoU, ExoT, and ExoY), and PA103 (cytotoxic; natively encodes ExoU and ExoT). This was done by the use of effector-null mutants with and without ExoS complementation in *trans* and of bacteria transformed with the pJNE05 T3SS reporter.

**FIG 4:**
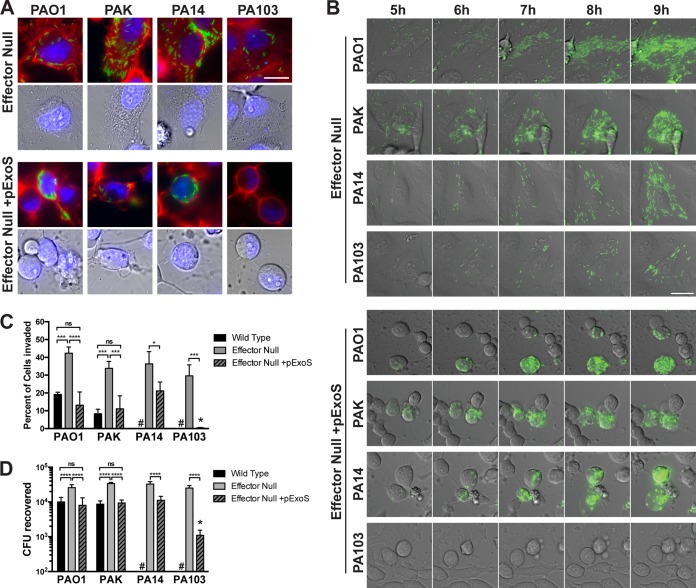
Internalization and outcomes of T3SS effector-null mutants of different P. aeruginosa strains with and without exogenous ExoS expression. (A) HeLa cells were infected with the indicated P. aeruginosa effector mutant strains transformed with T3SS-GFP reporter pJNE05 (green channel) and pUCP18-ExoS-HA (pExoS). Extracellular bacteria were killed with 200 µg/ml amikacin at 3 h. WGA (red channel) and Hoechst stain (blue channel) were added for 15 min and removed prior to imaging at 7 h postinfection. Bar, 25 µm. (B) HeLa cells were infected with the indicated strain transformed with T3SS-GFP reporter pJNE05 and pExoS. Extracellular bacteria were killed with 200 µg/ml amikacin at 3 h. Imaging began at 4 h postinfection and continued to be performed once per hour for 10 h in total. The corresponding times are indicated. Bar, 25 µm. (C) Time-lapse experiments were conducted as described for panel B, and Hoechst stain was added to visualize nuclei and expedite cell counting. Imaging began at 4 h postinfection and continued to be performed at 1-h intervals up to 10 h. The percentage of cells invaded and harboring replicating T3SS-positive bacteria was quantified over 3 experiments with 6 fields for each strain, mutant, or complemented mutant. #, not performed. Conditions were compared across strains by one-way ANOVA; a large single asterisk above the PA103 effector-null + pExoS designation indicates a significant difference from the results determined for other effector-null + pExoS strains. (D) HeLa cells were infected with the indicated strains with plasmids, and extracellular bacteria were eliminated with gentamicin at 3 h. At 4 h, cells were lysed with 0.25% (vol/vol) Triton X-100, and bacteria were enumerated by viable count. #, not performed. Conditions were compared across strains by one-way ANOVA; a large single asterisk above the PA103 effector-null + pExoS designation indicates a significant difference from the results determined for other effector-null + pExoS strains.

At 7 h postinfection, most cell-associated bacteria were T3SS positive, as verified using acquisition settings identical to those described for Fig. 2 in which the levels of T3SS-negative bacteria were just below the limit of detection (Fig. 4A). Without ExoS, the infected cells retained a relatively normal morphology over the experimental time period as typical for effector-null P. aeruginosa. When ExoS was expressed in *trans* under the control of its native promoter in plasmid pUCP18, HeLa cells underwent rounding and blebbing for all four strains in a similar time period (Fig. 4A). As expected, mutant PA103Δ*exoUT* + pExoS was not seen in intracellular locations (28). This contrasted with the results seen with the three other strains, which were all efficiently internalized despite the obvious impacts of pExoS on cell morphology. This included strain PA14, a cytotoxic strain that, like PA103, does not natively encode ExoS. Intracellular bacteria were found localized within membrane blebs (Fig. 4) in a manner similar to that seen with the invasive P. aeruginosa strains natively expressing ExoS (Fig. 3A).

Time-lapse live imaging was used to show the progression of P. aeruginosa replication within HeLa cells by the use of the T3SS reporter. After 3 h of infection, amikacin was added to kill extracellular bacteria, and live imaging captured the results hourly. Effector-null bacteria were T3SS positive while proliferating inside HeLa cells from 5 h to 9 h postinfection, with cells retaining normal morphology as expected (Fig. 4B). When these mutants harbored pExoS, HeLa cells were found to have rounded by 4 h as expected. Nevertheless, the bacteria continued to accumulate inside cells during the 5-h-to-9-h time window for all strains except PA103 (Fig. 4B). Time-lapse movies of effector-null PAO1 or effector-null PA103 complemented with pExoS or the empty vector (pUCP18) control infecting HeLa cells are shown in Movie S2a to f. Uninfected HeLa cells remained viable throughout the experiment (Movie S2g). Intervals of 10 min were used, and cell morphology was revealed by the use of differential interference contrast (DIC) overlaid with images representing the results obtained using the T3SS. While mutant PA103Δ*exoUT* + pExoS rarely invaded, cells took on the same morphology as other ExoS-secreting strains in the same time period.

10.1128/mBio.00668-18.4 MOVIE S2a  Time-lapse video microscopy movies of P. aeruginosa infection of HeLa cells over 20 h. Cells were infected with invasive strain PAO1 (Movie S2a) or its effector-null mutant PAO1Δ*exoSTY* (Movie S2b) or with mutant PAO1Δ*exoSTY* + pUCP18 (Movie S2c) or mutant PAO1Δ*exoSTY* + pExoS (Movie S2d). In other experiments, cells were infected with effector-null mutant PA103ΔexoUT + pUCP18 (Movie S2e) or mutant PA103ΔexoUT + pExoS (Movie S2f). Media were exchanged and amikacin was added at 3 h. Imaging began 4 h postinfection and continued to 20 h at 10-min intervals. Uninfected infected cells are also shown (Movie S2g). Download MOVIE S2a, MOV file, 34.8 MB. Copyright © 2018 Kroken et al. 2018 Kroken et al. This content is distributed under the terms of the Creative Commons Attribution 4.0 International license.

10.1128/mBio.00668-18.5 MOVIE S2b  See legend with Movie S2a. Download MOVIE S2b, MOV file, 46.6 MB. Copyright © 2018 Kroken et al. 2018 Kroken et al. This content is distributed under the terms of the Creative Commons Attribution 4.0 International license.

10.1128/mBio.00668-18.6 MOVIE S2c  See legend with Movie S2a. Download MOVIE S2c, MOV file, 47.5 MB. Copyright © 2018 Kroken et al. 2018 Kroken et al. This content is distributed under the terms of the Creative Commons Attribution 4.0 International license.

10.1128/mBio.00668-18.7 MOVIE S2d  See legend with Movie S2a. Download MOVIE S2d, MOV file, 38.2 MB. Copyright © 2018 Kroken et al. 2018 Kroken et al. This content is distributed under the terms of the Creative Commons Attribution 4.0 International license.

10.1128/mBio.00668-18.8 MOVIE S2e  See legend with Movie S2a. Download MOVIE S2e, MOV file, 38.9 MB. Copyright © 2018 Kroken et al. 2018 Kroken et al. This content is distributed under the terms of the Creative Commons Attribution 4.0 International license.

10.1128/mBio.00668-18.9 MOVIE S2f  See legend with Movie S2a. Download MOVIE S2f, MOV file, 34.5 MB. Copyright © 2018 Kroken et al. 2018 Kroken et al. This content is distributed under the terms of the Creative Commons Attribution 4.0 International license.

10.1128/mBio.00668-18.10 MOVIE S2g  See legend with Movie S2a. Download MOVIE S2g, MOV file, 37.9 MB. Copyright © 2018 Kroken et al. 2018 Kroken et al. This content is distributed under the terms of the Creative Commons Attribution 4.0 International license.

The percentage of HeLa cells invaded and the number of intracellular bacteria recovered in the experiments described above were quantified using multiple time-lapse movies such that 200 to 300 HeLa cells could be counted per experimental repeat (Fig. 4C). This approach yielded confidence in quantifying individual incidents of invasion over a large population, and the results complemented the gentamicin protection data. As expected, effector-null mutants were more extensively internalized than the respective wild-type strains; the levels of internalization were reversed by pExoS complementation. The percentage of HeLa cells invaded by mutant PA103Δ*exoUT* + pExoS was significantly lower than the percentage invaded by other effector-null mutants expressing pExoS (i.e., in each repeat performed with mutant PA103Δ*exoUT* + pExoS, only 0 to 2 invaded cells were identified among a total of 200 to 300 counted). A similar pattern of CFU recovery was found after gentamicin protection assays in which the medium was exchanged 3 h postinfection followed by 1 h gentamicin exposure (i.e., 4 h postinfection) to limit the time available for intracellular replication. Mutant PA103Δ*exoUT* + pExoS showed significantly reduced internalization compared to other complemented mutants (Fig. 4D).

### Cell infection with strain PA103 expressing ExoS blocks invasion of PAO1.

**FIG 5:**
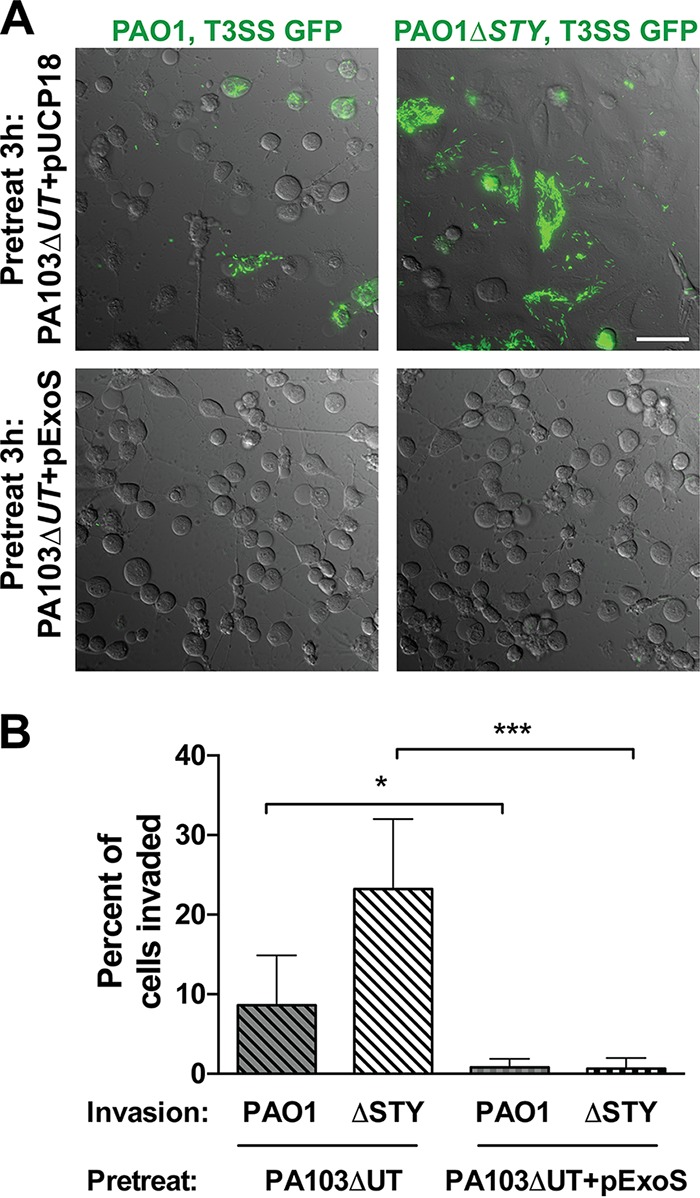
Prior exposure to ExoS results in blocked PAO1 internalization by HeLa cells. (A) HeLa cells were infected with mutant PA103Δ*exoUT* or mutant PA103Δ*exoUT* + pExoS for 3 h. PA103 mutants were eliminated by exposure to 200 µg/ml gentamicin for 15 min. Media were then removed and replaced with fresh medium without antibiotic. Strain PAO1 or mutant PAO1Δ*exoSTY* transformed with T3SS-GFP reporter pJNE05 (gentamicin resistant) was then added to the cells and incubated for 3 h. Extracellular PAO1 was killed by exposure to amikacin at 3 h. Imaging began at 7 h postinfection and continued to be performed at 1-h intervals until 16 h. Images represent the 11-h time point. Bar, 50 µm. (B) The percentage of cells invaded was quantified using time-lapse images from 2 to 300 cells counted in three independent experiments and analyzed by Student’s *t* test.

The results described above suggested that PA103 is unusual among P. aeruginosa isolates in how ExoS impacts its internalization. We next explored if the larger impact of pExoS on PA103 internalization than was seen with the other isolates was related to the impact on the host cell or to the bacterial capacity for internalization. This was done by first delivering ExoS to HeLa cells strictly by extracellular T3SS injection using strain PA103 and then studying its effect on internalization of strain PAO1 (Fig. 5). HeLa cells were infected with mutant PA103Δ*exoUT* containing pUCP18 or pExoS for 3 h, treated with gentamicin for 30 min to eliminate the presence of pretreatment mutant PA103Δ*exoUT*, and then infected with strain PAO1 or mutant PAO1Δ*exoSTY* expressing the T3SS reporter (confers gentamicin resistance). Pretreatment with mutant PA103Δ*exoUT* containing pUCP18 did not prevent the internalization of PAO1 or its effector-null mutant (Fig. 5A). However, their internalization was almost completely prevented when HeLa cells were preintoxicated with ExoS delivered by mutant PA103Δ*exoUT* (Fig. 5A). Quantitative analysis confirmed imaging observations, with effector-null PAO1 internalized by more epithelial cells than wild-type PAO1 as expected (Fig. 5B). These data show that PAO1 could not “override” ExoS intoxication by PA103 for invasion and suggested that the lack of internalization of mutant PA103Δ*exoUT* under conditions of expression of pExoS was related to its greater impact on the host cell population.

### FleQ modulates internalization.

PA103 lacks flagella (40) due to a point mutation in *fleQ*, a master regulator of flagellar expression and other virulence determinants (33, 38). Since flagellar motility and T3SS secretion are modulated inversely by Vfr in P. aeruginosa (41) and exhibit crosstalk (42), we hypothesized that the *fleQ* mutation was involved in the strong anti-internalization activity of mutant PA103Δ*exoUT* expressing pExoS.

**FIG 6:**
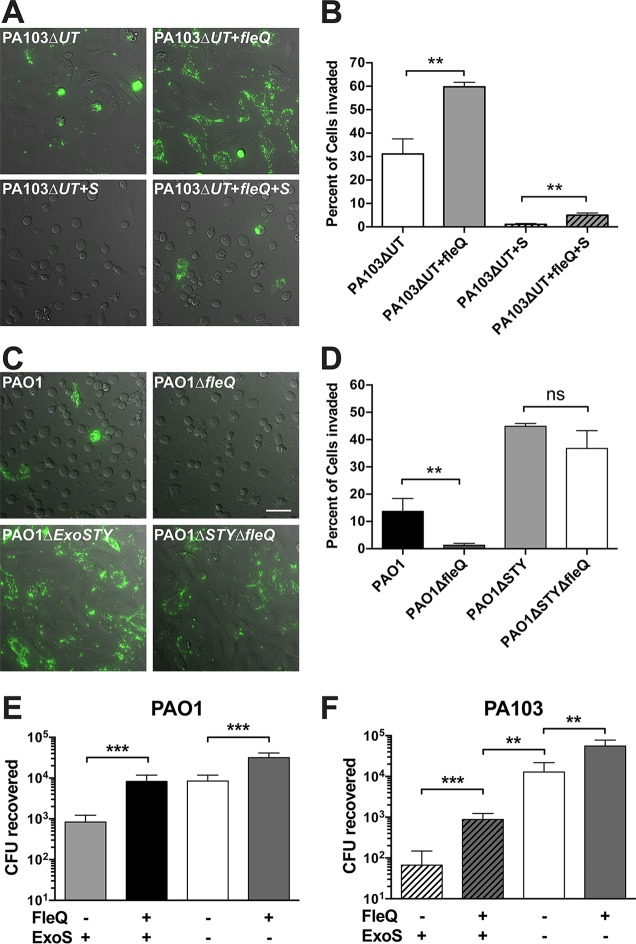
Functional *fleQ* is required for P. aeruginosa internalization in HeLa cells and opposes anti-internalization effects of ExoS. (A) Restoration of *fleQ* function in the PA103Δ*exoUT* mutant was accomplished with a V240G point mutation in mutant PA103Δ*exoUT*. HeLa cells were infected with the indicated strains, including transformation with pExoS. Extracellular bacteria were killed with 200 µg/ml amikacin at 3 h and imaged hourly from 4 h to 14 h postinfection; images are from the 10-h time point. (B) The percentage of cells showing bacterial internalization was determined, and the data in the indicated columns were compared using an unpaired *t* test. (C) The coding region of *fleQ* was deleted in strain PAO1 and mutant PAO1Δ*exoSTY*. HeLa cells were infected with the indicated strains transformed with T3SS-GFP reporter pJNE05 and imaged as described for panel A. Bar, 50 µm. (D) The percentage of cells showing bacterial internalization was determined as described for panel B, and data in the indicated columns were compared by unpaired *t* test. (E and F) Comparison of total levels of P. aeruginosa internalization in HeLa cells after infection with mutant PAO1Δ*exoSTY* (E) or mutant PA103Δ*exoUT* (F) with and without *fleQ* restoration and pExoS expression. Extracellular bacteria were eliminated with 200 µg/ml gentamicin at 3 h. At 4 h, total levels of internalized bacteria were enumerated by viable count. Data in the indicated columns were compared by unpaired *t* test.

The presence of a functional *fleQ* gene was restored in the genome of mutant PA103Δ*exoUT* as previously described (38). Controls confirmed that restored *fleQ* gene enabled the swimming motility of mutant PA103Δ*exoUT* (Fig. S2). *fleQ*-restored mutant PA103Δ*exoUT* was compared to uncorrected mutant PA103Δ*exoUT* with and without pExoS. Restoration of *fleQ* increased the percentage of invaded HeLa cells from <1% to about 5% for mutant PA103Δ*exoUT* expressing ExoS (*P* = 0.0027; Fig. 6A and B); that level was still low compared to results seen with other isolates expressing ExoS. Importantly, *fleQ* restoration also enhanced internalization of effector-null PA103 (without ExoS) to reach levels displayed by the other P. aeruginosa isolates. This showed that while *fleQ* promoted internalization, the mechanism was independent of the presence of ExoS.

10.1128/mBio.00668-18.2 FIG S2  Confirmation that *fleQ* mutants lack flagellum-based motility. Functional *fleQ* was restored in strain PA103Δ*exoUT* by generating the point mutation V240G. The *fleQ* open reading frame (ORF) was deleted in strain PAO1 and mutant PAO1Δ*exoSTY* by allelic exchange. Motility was determined by inoculating each strain in LB with 0.3% Bacto agar with colonies grown for 18 h at 30°C. Download FIG S2, TIF file, 79 MB. Copyright © 2018 Kroken et al. 2018 Kroken et al. This content is distributed under the terms of the Creative Commons Attribution 4.0 International license.

A clean genomic deletion of *fleQ* in strain PAO1 and mutant PAO1Δ*exoSTY* was made using allelic exchange, leaving only start and stop codons in place. The deletion was confirmed by PCR amplification of the region (not shown). Loss of swimming motility was confirmed visually using light microscopy (not shown) and an agar-based motility assay (Fig. S2). The *fleQ* mutation reduced internalization, as determined by counting the percentage of HeLa cells infected (Fig. 6C and D) and using gentamicin protection assays to quantify the total number of internalized bacteria (Fig. 6E). These assays confirmed that the impact of *fleQ* in PAO1 was independent of the presence of ExoS, as seen with mutant PA103Δ*exoUT*. Importantly, mutant PA103Δ*exoUT* remained ~10-fold less able to invade HeLa cells than PAO1 even when *fleQ* status was equilibrated, but that was the case only when ExoS was expressed (Fig. 6E and F). These data suggest two separate mechanisms for the unusually low internalization of mutant PA103Δ*exoUT* expressing ExoS, one involving *fleQ* mutation, the other modulating the impact of ExoS, and each reducing internalization ~10-fold to yield a combined 100-fold effect versus PAO1. This difference is potentially important since it causes mutant PA103Δ*exoUT* expressing pExoS to remain effectively extracellular whereas PAO1 exhibits both intracellular and extracellular lifestyles in epithelial cell infection.

### The impact of FleQ involves FlhA and FliC.

**FIG 7:**
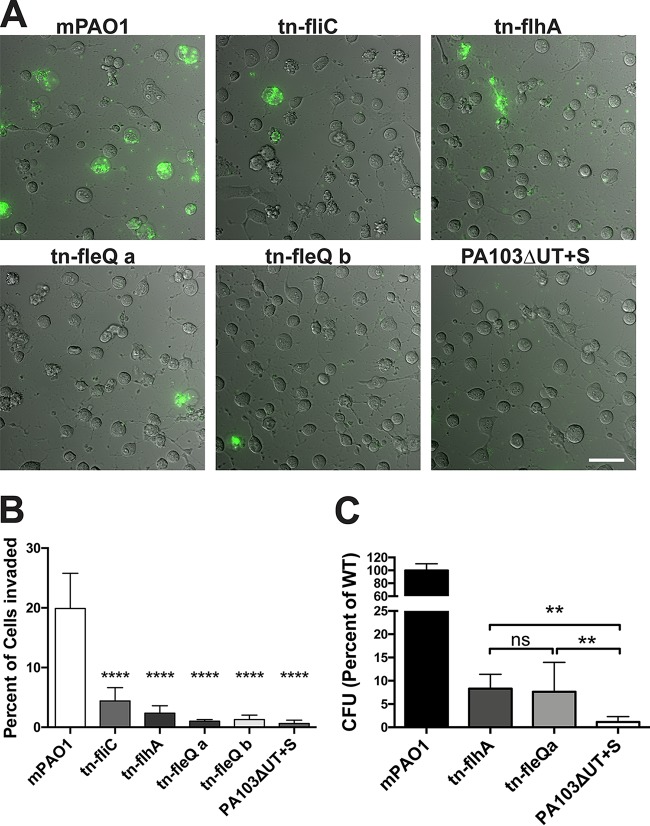
P. aeruginosa motility mutants show reduced internalization by HeLa cells but still induce cell rounding. (A) The parent mPAO1 strain and the indicated transposon mutants were transformed with T3SS-GFP reporter pJNE05 and used to infect HeLa cells. Extracellular bacteria were killed with 200 µg/ml amikacin at 3 h. Imaging began at 4 h postinfection and continued to be performed hourly for 10 h. Bar, 50 µm. (B) Percentages of cells showing P. aeruginosa internalization were quantified using the results depicted in panel A and analyzed by one-way ANOVA compared to mPAO1 results. (C) The quantity of internalized motility mutants was determined by gentamicin protection assay as a percentage of wild-type mPAO1 data. Extracellular bacteria were eliminated with 200 µg/ml gentamicin at 3 h; total levels of internalized bacteria were enumerated by viable count at 4 h. Data in the indicated columns were compared by one-way ANOVA.

FleQ modulates flagellar assembly. However, it has also been shown to regulate other virulence determinants and may indirectly influence T3SS secretion (42, 43). To explore the role of flagellar assembly, we obtained *fleQ*, *flhA*, and *fliC* mutants from the P. aeruginosa transposon library of PAO1 (44). After PCR verification of the mutants and confirmation of lack of swimming motility using light microscopy (data not shown), these were transformed with the T3SS reporter plasmid. Internalization of the mutants was compared to internalization of the wild-type strain from the transposon library (mPAO1) and also to internalization of mutant PA103Δe*xoUT* + pExoS by the use of time-lapse imaging and amikacin protection (Fig. 7). While mPAO1 invaded similarly to the PAO1 version used in other experiments, all of the mutants invaded significantly fewer HeLa cells, evidenced by quantification of the percentage of cells containing internalized bacteria from time-lapse videos (Fig. 7A and B). Gentamicin protection assays provide greater resolution for comparisons between low numbers due to assessing the entire well of adherent HeLa cells (which represents ~2e−5 cells), whereas our time-lapse microscopy invasion assay was limited to a few hundred cells. This assay revealed no significant differences between the levels seen with the *flhA* mutant and the *fleQ* mutant of PAO1; however, both levels were significantly different from the low level of invasion by mutant PA103ΔexoUT + pExoS (Fig. 7C). HeLa cell morphology was impacted in equal manners by all mutant strains and mPAO1, suggesting that ExoS was efficiently delivered into host cells in all instances. Where invasion did occur, T3SS-positive bacteria were visible inside blebs at levels similar to those seen with the wild-type strains, showing that *fleQ* and *flhA* and flagella were not needed for subsequent steps of intracellular trafficking to bleb niches that are dependent on ExoS and other components of the T3SS.

### Under inducing conditions, PA103 activates the T3SS more robustly than other strains.

**FIG 8:**
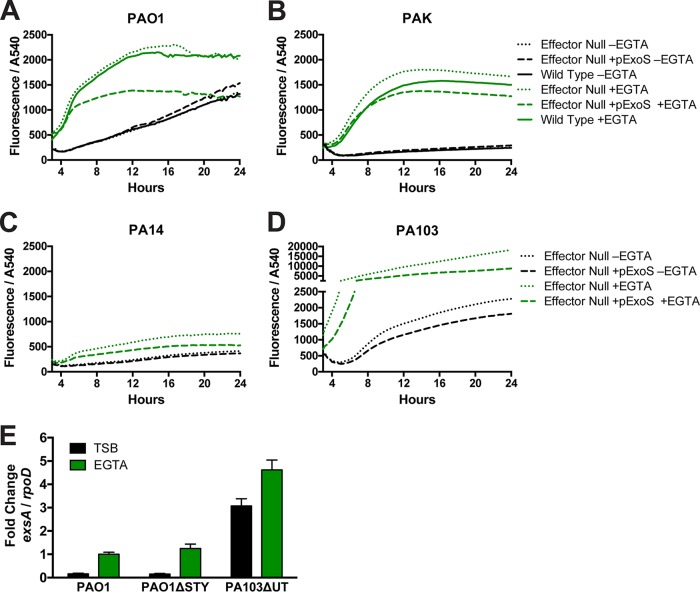
Fluorescence intensity of the T3SS-GFP reporter in invasive and cytotoxic P. aeruginosa strains during growth under T3SS-inducing conditions. (A to D) Absorbance (OD_540_) was used to measure growth of invasive strains (A and B) or cytotoxic P. aeruginosa strains (C and D) in TSB containing 100 mM monosodium glutamate (MSG) and 1% glycerol. EGTA (2 mM) was added to media for T3SS induction. Levels of absorbance at 540 nm and GFP fluorescence were measured every 15 min. Data represent levels of fluorescence intensity normalized to absorbance over 24 h of growth determined in a representative experiment performed with four technical replicates and were averaged for each strain and condition. Data from PA103 (panel D) are shown on a discontinuous scale to accommodate the greater levels of fluorescence intensity measured. (E) RNA was isolated from indicated strains grown 8 h in TSB or in inducing media containing EGTA (2 mM). Expression of *exsA* relative to that of the *rpoD* housekeeping gene was determined using real-time quantitative reverse transcription-PCR (qRT-PCR), and the results showed greater *exsA* expression in mutant PA103Δ*exoUT* consistent with greater T3SS-GFP reporter intensity.

The data presented above showed that, while the *fleQ* mutation in PA103 contributes to its reduced internalization capacity, other factors become equally important when ExoS is expressed. Thus, we tested the hypothesis that this relates to differences in T3SS expression among isolates. We quantitatively compared the levels of T3SS reporter gene expression in the four P. aeruginosa strains under conditions that were noninducing (lacking EGTA) and inducing (2 mM EGTA) with respect to T3SS gene expression. While all four isolates responded to inducing conditions, PA103 yielded much greater T3SS reporter expression than all of the other strains (Fig. 8D; note the *y* axis scale break). This was unrelated to it being a cytotoxic strain; compared to the other cytotoxic strain, PA14 (Fig. 8C), there was a striking ~10-fold difference in the response, and that difference was not a consequence of bacterial growth rates. Indeed, PA14 was less responsive than invasive isolates, which was somewhat surprising given our observations in cell infection experiments for intracellular bacteria (Fig. 4; all GFP channels are scaled identically). Considering the presence of previously reported feedback mechanisms (45), the reduced fluorescence observed with all effector-null mutants transformed with pExoS (green dashed lines) compared to the respective nontransformed controls was expected. With or without pExoS, however, the data suggested that effector-null PA103 differed from the other effector-null strains with respect to the high levels of T3SS expression observed under inducing conditions *in vitro*. To account for possible differences in plasmid copy numbers or limits of GFP folding where the T3SS-GFP reporter was used as a proxy for ExsA-induced transcription, mutant PA103Δ*exoUT* was compared to strain PAO1 and mutant PAO1Δ*exoSTY* using real-time quantitative PCR. RNA was isolated from bacteria grown for 8 h, and transcripts were normalized to the activity of the *rpoD* housekeeping gene. The relative ratios resulting from comparisons between strain PAO1, its effector-null mutant, and mutant PA103Δ*exoUT* mirrored those found in the growth assay measuring T3SS-GFP reporter fluorescence, supporting the premise that the reporter reflects *exsA* transcription.

### Hyperinducibility of T3SS expression in PA103 is unrelated to *fleQ* mutation.

**FIG 9:**
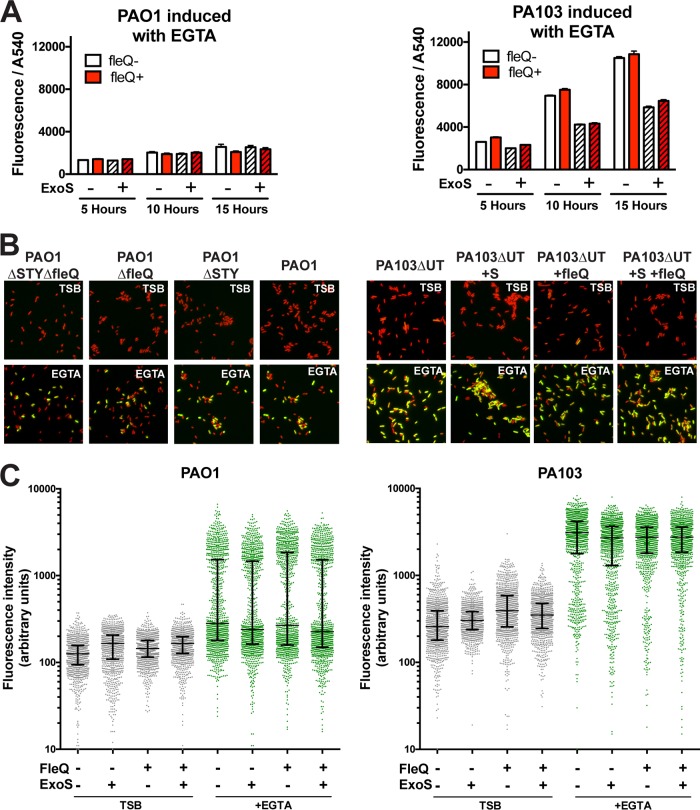
(A) Absorbance (OD_540_) was used to measure the growth of P. aeruginosa strain PAO1 or mutant PA103Δ*exoUT* in TSB containing 100 mM MSG and 1% glycerol as well as EGTA (2 mM) for T3SS induction. Levels of absorbance at 540 nm and GFP fluorescence were measured, and the results seen at the 5-, 10-, and 15-h time points are shown as bar graphs. (B) The indicated strains expressing the T3SS reporter were grown in TSB or in inducing media up to 8 h, fixed with paraformaldehyde, stained with propidium iodide (PI), and imaged. PI is indicated in red and GFP in green. (C) The levels of GFP intensity of individual bacteria were quantified from the images represented in panel B. The bars show the medians and interquartile ranges of the data from the indicated populations.

Mutants of *fleQ* were tested with the T3SS reporter in a growth assay using inducing media to determine if changes to T3SS regulation were occurring as a result of introducing this mutation; if so, that could influence invasion. Under conditions of growth in inducing media, the levels of fluorescence intensity of the total population normalized to optical density were similar whether or not functional *fleQ* was present for both strain PAO1 and strain PA103 at the three time points tested (Fig. 9A).

Bacteria were removed from the growth assay at 8 h, fixed in paraformaldehyde, and examined by microscopy. Propidium iodide was used to counterstain all bacterial bodies to facilitate software detection, and GFP intensity was measured (Fig. 9B). Figure 9C shows data from 1,000 individual bacteria from multiple fields, capturing a bimodal distribution of GFP intensity data under conditions of induction with EGTA as previously reported (46, 47). The median and the interquartile range are shown as bars to highlight the distribution of the population. The status of *fleQ* had little impact on the portion of bacteria that became T3SS positive. However, in comparisons of the two strains, less than half of the EGTA-induced PAO1 bacteria became T3SS positive but greater than 75% of EGTA-induced PA103 bacteria turned on the reporter; the number of T3SS-positive bacteria was consistent with previous studies that utilized fluorescent sorting technology on strain PA103 (46, 47) and with reported observations (48) from Kazmierczak’s research group (Yale School of Medicine). Individual PA103 bacteria scored slightly higher in fluorescence overall than PAO1 bacteria (6,649 fluorescent units for PAO1 versus 8,275 for mutant PA103Δ*exoUT*), but the proportion of each population determined on the basis of T3SS status explains the overall higher intensity of GFP in the PA103 population (Fig. 8). Importantly, this property of PA103 was not modulated by *fleQ* status or even impacted greatly by ExoS expression. Instead, this distribution of T3SS-positive versus T3SS-negative bacteria points to a second feature of PA103 which we term "hyperinducibility," which associates with unique T3SS dynamics upon host cell contact.

## DISCUSSION

P. aeruginosa continues to be classified as an extracellular pathogen by many in the field, despite a plethora of publications suggesting that it invades and replicates in a variety of cell types. Contributing to this dichotomy, methods commonly used to demonstrate intracellular localization can have caveats. Non-cell-permeative antibiotic protection assays can provide misleading data if extracellular bacteria are in a resistant (e.g., biofilm) state or if they are trapped in an extracellular location that does not allow antibiotic access. When this method works as intended, it provides only a single measure of total intracellular bacteria in a sample. Standard methods for imaging also suffer from limitations with respect to convincingly distinguishing intracellular from extracellular bacteria, with snapshots not necessarily representing frequency or event sequences. Contributing to the skepticism surrounding the ability of P. aeruginosa to invade epithelial cells, T3SS effectors encoded by P. aeruginosa strains prevent internalization in HeLa cells and yet promote intracellular survival in corneal and bronchial epithelial cells.

Here, we devised advanced methods for studying P. aeruginosa localization during cell infection and used them in tandem with a T3SS reporter to test hypotheses for reconciliation with the published literature. Possibilities considered included differences between cell types and bacterial strains and that misleading data might have been published about invasion due either to low numbers of epithelial cells being sampled for imaging or to the fact that the non-cell-permeative antibiotics used to kill extracellular bacteria were not entirely effective.

After showing that HeLa cells were at least as susceptible as corneal epithelial cells to invasion and intracellular survival by P. aeruginosa, we turned our attention to strain-related variables. The results showed that PA103, the laboratory strain most commonly used to study the impact of T3SS effectors on host cell biology and regulation of T3SS gene expression, was unusual among P. aeruginosa isolates in having a low capacity for epithelial invasion when expressing ExoS without other T3SS effectors. This was shown to be related in part to its known *fleQ* mutation, which renders it nonmotile and lacking the invasion factor FlhA. However, there were also *fleQ*-independent impacts on invasion for PA103 that were detected only when ExoS was expressed. This was not related to its being a cytotoxic strain normally lacking ExoS, as another cytotoxic strain (PA14) behaved similarly to the invasive isolates when it encoded ExoS. Instead, the data showed that PA103 differed from the other three strains in being T3SS hyperinducible. That was demonstrated *in vitro* under inducing conditions. It was also observed visually for extracellular bacteria in cell infection experiments, with the T3SS being mostly active for PA103 and mostly not active for the three other isolates examined. Nevertheless, intracellular bacteria strongly expressed the T3SS for all four isolates, including daughter bacterial cells, after intracellular replication.

Experiments using various methods, including high-resolution confocal imaging showing clearly delineated epithelial cell membranes, confirmed that the bacteria surviving incubation in gentamicin were intracellular rather than being present in potentially resistant extracellular biofilms or hiding under epithelial cells. Further, internalization was not a rare event, with ~20% of cells invaded, a number correlating well to the results of gentamicin protection assays and explaining those findings on a per-cell basis. Within HeLa cells, as within corneal and bronchial epithelial cells (16, 30, 32), intracellular bacteria expressing ExoS replicated rapidly within plasma membrane blebs and in the cytosol, with formation and trafficking to blebs being dependent on the presence of ExoS. Further, when mutant PA103Δ*exoUT* expressing ExoS did invade a cell (doing so with ~1 or 2 of ~300 epithelial cells sampled in each biological repeat), it also demonstrated this phenotype. Thus, the unique intracellular survival strategy exhibited by P. aeruginosa under conditions of expression of ExoS involving formation and trafficking to blebs does not appear to be specific to particular epithelial cell types or to P. aeruginosa strains.

Two separate outcomes suggested that T3SS/ExoS hyperinducibility and the lack of internalization by mutant PA103Δ*exoUT* expressing ExoS were directly linked mechanistically. First, mutant PA103Δ*exoUT* blocked internalization of another strain that otherwise invaded efficiently (PAO1) but did so only when it expressed ExoS. Second, mutant PA103Δ*exoUT* without ExoS was internalized similarly to other P. aeruginosa isolates when matched for *fleQ*.

FleQ is a member of the NtrC subfamily of transcriptional activators that regulates flagellar synthesis and export and polar localization (43, 49). The *fleQ* mutation in PA103 contributes to its reduced capacity to invade mammalian cells (an ~10-fold reduction whether or not ExoS was expressed). That was an expected result, considering that *fleQ* mutants lack the flagella important for motility and adhesion function (50) and that our previous work showed that two flagellum-related genes (*fliC* [encoding flagellin] and *flhA* [encoding the flagellum export apparatus; homologue of Salmonella enterica *invA*]) promote P. aeruginosa internalization into corneal epithelial cells (51). Here, this was also shown for HeLa cells. Interestingly, when *fleQ*-related (*fleQ*, *flhA*, or *fliC*) mutants were internalized, albeit in smaller numbers, the sequence of events that followed was exactly the same as for wild-type PAO1, with bacteria surviving intracellularly while forming and trafficking to membrane blebs. Thus, P. aeruginosa invasion and subsequent events do not require these flagellum-related genes, even though their presence increases the probability of internalization.

A previous study identified cross talk between flagellar assembly and the T3SS, showing that overexpression of the T3SS activator ExsA decreased motility and that *fliC* mutants produced greater quantities of ExoS (42). In the present study, there was little evidence for functional cross talk between these systems. For example, *fleQ* had no impact on the percentage of T3SS-induced bacteria for either PA103 or PAO1 under inducing conditions, irrespective of ExoS expression (Fig. 8). Further, the flagellum-related (*fleQ*, *flhA*, and *fliC*) mutants universally rounded HeLa cells by 3 h, a well-established metric for ExoS delivery (52), showing that the type of cell contact required for T3SS induction and effector delivery to allow the rounding phenotype also did not require these genes (Fig. 2 and 4).

Time-lapse imaging of P. aeruginosa invading epithelial cells expressing the T3SS reporter revealed GFP expression in bacteria that was apparently occurring mostly after internalization (see Movie S1 in the supplemental material). T3SS induction after internalization might explain why invasive strains encoding ExoS, and other T3SS effectors with anti-internalization activity, are internalized by a significant number of epithelial cells, and it aligns well with the demonstrated role of ExoS in intracellular survival. It would also fit with data in this study showing that mutant PA103Δ*exoUT* hyperinducibility occurred *in vitro* and extracellularly in our imaging experiments, explaining why fewer epithelial cells than normal internalized bacteria that were fully capable of invading cells when not expressing ExoS. While the *exsA* and GFP expression data were consistent, a remaining limitation was that the exact timing of T3SS expression with respect to cell entry was difficult to definitively determine. The observation of cell rounding (dependent upon ExoS delivery) prior to detection of GFP is suggestive of a delay between T3SS gene induction and the ability to detect GFP from the reporter. How much ExoS is required to prevent internalization, as well as the proportions encoded by intracellular versus extracellular bacteria, is also not clear at present and is likely to vary depending on conditions. However, data shown in this study suggest the potential for intracellular bacteria to make T3SS effectors and raise the possibility that extracellular delivery might not be necessary for cellular intoxication. Indeed, we previously reported that ExoS-dependent P. aeruginosa intracellular replication can occur independently of the T3SS translocon (29, 53). With respect to pathogenesis, it could be advantageous for bacteria to deliver effectors from either location depending on the prevailing situation, thereby enabling them to occupy both intracellular and extracellular spaces and still manipulate host cell function. Given that the mammalian cell cytosol contains less than 100 nM Ca^2+^ except under conditions of undergoing calcium-mediated signaling (54), the notion that T3SS expression could be induced inside mammalian cells aligns with our current understanding of how this system is regulated via calcium chelation *in vitro* (14, 55, 56). Indeed, we showed in a recent study that epithelial cell lysates could induce expression of a modified form of ExoS, a finding that might involve low levels of Ca^2+^ and/or other cytosolic factors (57).

In addition to considering the location of T3SS induction, our data suggest that bistability of T3SS expression is also an important factor to consider in understanding P. aeruginosa interaction with host cells. Bistability refers to differences in T3SS gene expression in a single population exposed to the same inducing stimulus (58). Indeed, we previously hypothesized that T3SS bistability might explain why only some intracellular P. aeruginosa escaped acidified vacuoles or why P. aeruginosa is internalized while encoding antiphagocytic effectors (29), the latter being the focus of this study. The PA103 hyperinducibility shown here could reflect a difference in the bistability “set point” in this strain compared to others, causing the significant shift toward T3SS-positive bacteria in the effector-null PA103 population at any given time point, and the absence of their internalization in the presence of ExoS. As such, these data support a role for T3SS bistability in influencing the outcome of P. aeruginosa-host cell interactions.

Together, these data convincingly establish that P. aeruginosa enters epithelial cells, with subsequent intracellular survival and replication occurring in both the cytosol and bleb niches, and that its entry is not restricted with respect to the strain type or epithelial cell type. The results also show that the percentage of epithelial cells invaded can be significant and is influenced by *fleQ*-related and T3SS-related mechanisms that function independently of one another. While the data show strong intracellular T3SS expression, more work is needed to determine the relative levels of importance of extracellular induction versus intracellular induction and of T3SS bistability under each circumstance. It will also be important to determine the conditions under which these events occur *in vivo* and how they contribute to tissue pathology and disease outcomes.

## MATERIALS AND METHODS

### Bacterial strains, plasmids, and mutants.

Table 1 shows the P. aeruginosa strains and plasmids used. Mutants from the P. aeruginosa PAO1 transposon mutant library were confirmed by the use of recommended primers. P. aeruginosa was transformed by electroporation as follows: log-phase cultures were suspended in 300 mM sucrose, combined with 100 ng of plasmid in a 0.2-cm-gap cuvette and pulsed (200 Ω, 25 µF, 2.5 kV) for 2 s, grown for 1 h in LB media, and plated on tryptic soy agar (TSA) plates with antibiotic selection (200 µg/ml carbenicillin or 200 µg/ml gentamicin [20 µg/ml gentamicin for PA14]).

**TABLE 1:** Strains and plasmids used in this study

Strain or plasmid	Description	Source or reference
Strains		
PAO1	Wild type	Arne Riestch (Case Western Reserve University)
PAO1Δ*exsA*	*exsA* mutant	Arne Riestch (Case Western Reserve University)
PAO1Δ*exoSTY*	*exoS*, *exoT*, and *exoY* mutant	Arne Riestch (Case Western Reserve University)
PAO1Δ*fleQ*	*fleQ* mutant	This study
PAO1Δ*exoSTY*Δ*fleQ*	*exoS*, *exoT*, *exoY*, and *fleQ* mutant	This study
PAK	Wild type	Joanne Engel (University of California, San Francisco)
PAKΔ*exoSTY*	*exoS*, *exoT*, and *exoY* mutant	Joanne Engel (University of California, San Francisco)
PA103Δ*exoUT*	*exoU* and *exoT* mutant	Dara Frank (Medical College of Wisconsin)
PA103Δ*exoUT+fleQ*	*exoU* and *exoT* mutant with restored *fleQ* (T719G)	This study
PA14Δ*exoUTY*	*exoU*, *exoT*, and *exoY* mutant	Gerald Pier (Harvard Medical School)
mPAO1	Transposon mutant library parent	PAO1 transposon mutant library (44)
mPAO1 tn-*fliC*	PW8407 fliC-B03::ISphoA/hah	PAO1 transposon mutant library (44)
mPAO1 tn-*flhA*	PW3636, flhA-E11::ISlacZ/hah	PAO1 transposon mutant library (44)
mPAO1 tn-*fleQ* a	PW2981, fleQ-G06::ISphoA/hah	PAO1 transposon mutant library (44)
mPAO1 tn *fleQ* b	PW2980, fleQ-C04::ISphoA/hah	PAO1 transposon mutant library (44)
		
Plasmids		
pUCP18	P. aeruginosa expression vector	Joseph Barbieri (Medical College of Wisconsin)
pExoS (pUCP18 ExoS-HA)	ExoS expression vector	Joseph Barbieri (Medical College of Wisconsin)
pJNE05	T3SS-GFP reporter	Timothy Yahr (University of Iowa) (35)
p67T1	dTomato expression vector	John Singer (University of Maine) (36)
pEXG2	Integrating suicide plasmid	Arne Riestch (Case Western Reserve University)

Clean deletion of *fleQ* in PAO1 was performed by allelic exchange as follows: 500-bp regions flanking *fleQ* were amplified (see Table 2 for primers) and combined by overlap extension, leaving the start and stop codon of *fleQ* intact. This fragment was cloned into pEXG2 using EcoR1 and HindIII restriction sites, and P. aeruginosa was transformed by conjugation with Escherichia coli SM10 on LB agar and counterselected on 5% sucrose LB agar. Restoration of functional *fleQ* (T719G [yielding a V240G amino acid substitution]) in mutant PA103Δ*exoUT* was accomplished by amplifying an internal 1,440-bp region of *fleQ* from mutant PA103Δ*exoUT* genomic DNA and performing QuikChange Lightning mutagenesis (see Table 2). Functional *fleQ* was complemented by allelic exchange as described above for PAO1. Mutants were confirmed by sequencing and motility tested in 0.3% Bacto agar LB plates.

**TABLE 2:** Primers used in mutagenesis or qRT-PCR^a^

Primer name	Sequence	Source or reference
*fleQ* upstream F	5′-GATAAAGCTTATCGGTGAGCTGGATCAGGTC-3′	This study
*fleQ* upstream R	5′-GTTGCGAAACGACCTGTCACATTTTGATCAGCTGCC-3′	This study
*fleQ* downstream F	5′-GGCAGCTGATCAAAATGTGACAGGTCGTTTCGCAAC-3′	This study
*fleQ* downstream F	5′-CATGGAATTCCCTCGCGCGGAGCGAAGCAGC-3′	This study
PA103 *fleQ* F	5′-GATAAAGCTTCTGGCCAGTTGGGACGAGTACCTG-3′	This study
PA103 *fleQ* R	5′-CATGGAATTCGTCGTCGAGGGCCTGCTGGATC-3′	This study
*fleQ* T719G F	5′-CTGGCCAACGGCGGCACCCTGTTCCTC-3′	This study
*fleQ* T719G R	5′-CTGGCCAACGGCGGCACCCTGTTCCTC-3′	This study
RT *rpoD* F	5′-GGGCGAAGAAGGAAATGGTC-3′	59
RT *rpoD* R	5′-CAGGTGGCGTAGGTGGAGAA-3′	59
RT *exsA* F	5′-TCAAGGGGTTGAAGGAATTG-3′	This study
RT *exsA* R	5′-CAGCTTCCACTCGTTGAGGT-5′	This study

a qRT-PCR, quantitative reverse transcription-PCR.

### Cell culture.

HeLa cells were cultured in Dulbecco’s modified Eagle’s medium (DMEM) mixed with 10% fetal bovine serum. Human telomerase-immortalized corneal epithelial cells (hTCEpi) were maintained in KGM-2 media (0.15 mM calcium) (Lonza) in an undifferentiated state and were differentiated by the use of 1.15 mM calcium. NuLi-1 cells (ATCC CRL-4011) were maintained in bronchial epithelial cell growth medium (BEGM) (Lonza). Cells were seeded onto ibidi 8-well poly-l-lysine-coated polymer microslides for live imaging or 24-well tissue culture dishes for gentamicin protection experiments.

### Infection of cultured cells.

P. aeruginosa cells were grown as a lawn on TSA containing selective antibiotics overnight at 37°C. Bacteria were suspended in sterile phosphate-buffered saline (PBS) by gentle pipetting. Multiplicities of infection (MOIs) were calculated using an *A*_540_ of 1 (4 × 10^8^ CFU). An MOI of 10 was used for all experiments, since it was in the linear range of PAO1 uptake (not shown). Where indicated, at 3 h postinfection, medium was replaced with fresh media containing 200 µg/ml amikacin or gentamicin to kill extracellular bacteria or containing 25 µg/ml ofloxacin to kill both extracellular and intracellular bacteria. After antibiotic treatment, internalized bacteria were enumerated by lysing epithelial cells using Triton X-100 (0.25%) and performing viable counts using PBS for dilution and MacConkey agar plates. Inocula were confirmed via the same method.

### *In vitro* bacterial growth curves.

Medium (100 µl) was added to a 96-well plate and inoculated with ~2 × 10^6^ CFU bacteria. Tryptic soy broth (TSB) supplemented with 100 mM monosodium glutamate and 1% glycerol was used as a neutral growth medium. EGTA (2 mM) (pH adjusted to 7.0 with NaOH) was added for T3SS induction. Optical density at 540 nm (OD_540_) and GFP fluorescence were read every 15 min on a BioTek Synergy HTX plate reader warmed to 37°C with shaking between readings. *In vitro* growth curves or bar graphs show data from a representative experiment performed with an average of three technical replicates. Experiments were repeated three times.

### Live-cell labeling.

Live cells were labeled with 2.5 µg/ml wheat germ agglutinin (WGA)–Alexa Fluor 647 (Thermo Fisher) for 10 min to visualize membranes. Media were carefully replaced to remove unbound WGA without lifting rounded cells followed by immediate imaging. To visualize nuclei, Hoechst stain (Thermo Fisher) was added to cells 20 min before imaging.

### Imaging of fixed bacteria.

Bacteria were grown with shaking for 8 h in a 96-well plate at 37°C. The culture was combined with an equal volume of 4% paraformaldehyde–PBS for 15 min. Bacteria were centrifuged at 14,000 × *g* for 5 min, suspended in PBS for 10 min with propidium iodide, washed again, and suspended in ultrapure water. The suspension was spotted onto a coverslip, dried, and mounted on slides with ProLong diamond mounting media. Five fields were imaged with identical acquisition parameters for each condition, and the experiment was repeated twice.

### Microscopy.

Live images and time-lapse videos were captured on a Nikon Ti-E inverted wide-field fluorescence microscope equipped with a Lumencor SpectraX illumination source and an Okolab Uno-combined-controller stage top incubation chamber to maintain heat, humidity, and 5% CO_2_. Time-lapse images were captured using a CFI Plan Apo Lambda 40× air objective, and still images were captured using a CFI Plan Apo Lambda 60× oil objective, each equipped with differential interference contrast (DIC). Hoechst stain was added to facilitate automated counting of epithelial cells. For time-lapse imaging, six fields were chosen randomly. Confocal images were acquired on an Olympus Fluoview FV1000 upright laser scanning confocal microscope using a water immersion 60× objective.

### Image analysis.

Enumeration of blebbing cells was determined visually. The average fluorescence intensity of cell-associated bacteria was measured using ImageJ. Ovals were drawn around overlapping bacteria, and the average fluorescence intensity of the area was measured. Total epithelial cells were counted using the first frame of Hoechst imaging, and raw images of nuclei were processed using the following ImageJ commands as a macro: Gaussian Blur 5 > Auto Threshold with black background > Convert to mask > Watershed > Analyze Particles. Invaded cells were counted using time-lapse micrographs to identify cells harboring T3SS-positive bacteria that had divided at least once and that had migrated within the boundary of the cell. Numbers of invaded epithelial cells expressed as a percentage of total cells were totaled from six fields, and bar graphs show the percentages of cells invaded as determined using means and standard deviations of results from at least three independent experiments. For measuring the GFP intensity of fixed bacteria, regions of interest (ROIs) were drawn to avoid measuring areas in which bacteria were clumped. Images were adjusted for noise with the Smooth process in ImageJ, individual bacteria were detected in the propidium iodide channel using Find Maxima, and measurements of individual points were taken in the GFP channel. The value corresponding to the average background GFP intensity was subtracted.

### RNA extraction and real-time quantitative RT-PCR.

Cultures (10 ml) were inoculated with 1e−8 CFU of strain PAO1, mutant PAO1Δ*exoSTY*, and mutant PA103Δ*exoUT* and grown for 8 h. Cultures were centrifuged at 7,000 × *g* for 4 min and pellets suspended in 1.2 ml of TRIzol reagent (Invitrogen). RNA was purified with a Direct-zol RNA MiniPrep kit (Zymo Research), followed by batch digestion performed with recombinant DNase I (Roche), and was purified again with the Direct-zol kit. The cDNA was synthesized with an iScript cDNA synthesis kit (Bio-Rad). Real-time quantitative PCR was performed on 20 ng of cDNA, and amplification was performed using LightCycler 96 DNA Sybr green I master mix (Roche). Specific primers for *rpoD* were obtained from the literature (59), and primers for *exsA* were designed for this study. Reactions were monitored using a Roche LightCycler 96 instrument. Transcript amounts were standardized to transcription of *rpoD* (relative quantification using the quantification cycle [ΔΔ*C*_*q*_] method). Experiments were repeated three times; results represent a representative replicate with technical error data.

### Statistical analyses.

Data are shown as means with standard deviations except for the data expressing the GFP intensity of individual bacteria, which are shown as medians with interquartile ranges. The significance of differences between groups was determined with an unpaired Student’s *t* test for data determined under two conditions or with one-way analysis of variance (ANOVA) with Tukey *post hoc* analysis for data determined under three or more conditions. GraphPad Prism 6 was used for calculations. In all figures, *P* values of ≤0.05 are represented by a single asterisk (*), *P* values of ≤0.01 are represented by a two asterisks (**), *P* values of ≤0.005 are represented by a three asterisks (***), *P* values of ≤0.001 are represented by a four asterisks (****), and “ns” denotes that the results were not statistically significant.
