# Supplementary figures and images for: The Impact of ExoS on Pseudomonas aeruginosa Internalization by Epithelial Cells Is Independent of fleQ and Correlates with Bistability of Type Three Secretion System Gene Expression
Source: mBio. 2018 May 1;9(3):e00668-18. doi: 10.1128/mBio.00668-18 (PMC5930308; doi:10.1128/mBio.00668-18)

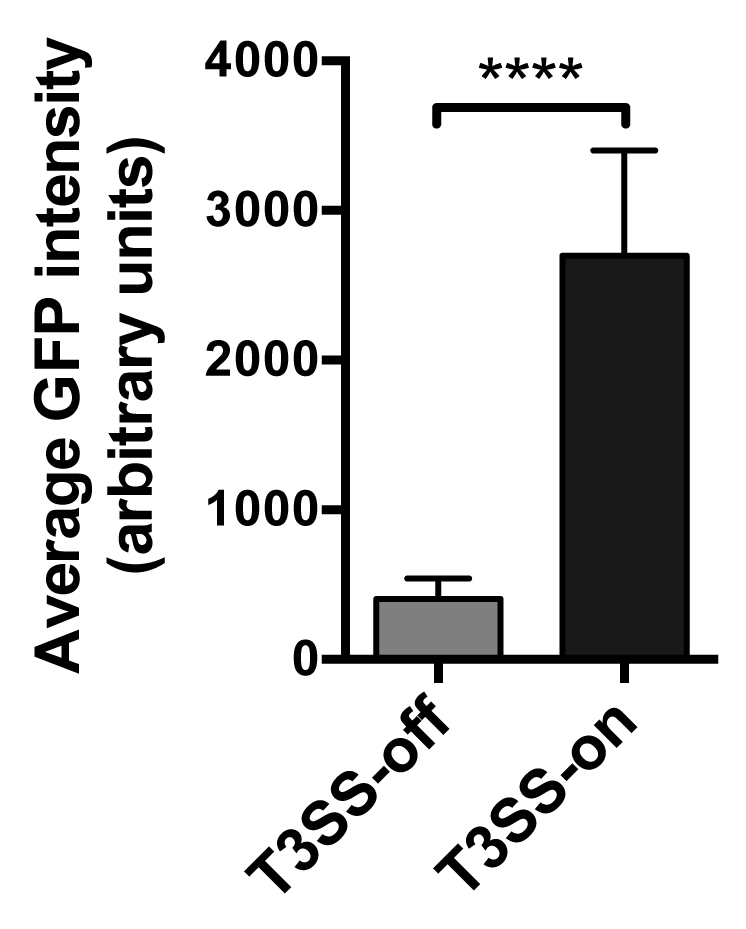

Supplement: FIG S1 [file mbo002183856sf1.tif]
